# Case report: small cell transformation and metastasis to the breast in a patient with lung adenocarcinoma following maintenance treatment with epidermal growth factor receptor tyrosine kinase inhibitors

**DOI:** 10.1186/s12885-016-2623-4

**Published:** 2016-08-03

**Authors:** Quan Lin, Guo-ping Cai, Kai-Yan Yang, Li Yang, Cheng-Shui Chen, Yu-Ping Li

**Affiliations:** 1 Department of Pulmonary Medicine, The First Affiliated Hospital of Wenzhou Medical University, Wenzhou, Zhejiang 325015 China; 2 Department of Pathology, Yale University School of Medicine, New Haven, Connecticut USA; 3 Department of Pathology, The First Affiliated Hospital of Wenzhou Medical University, Wenzhou, Zhejiang 325015 China

**Keywords:** Adenocarcinoma, Small cell lung cancer, Metastatic breast tumor, Epidermal growth factor receptor, Tyrosine kinase inhibitor

## Abstract

**Background:**

Breast metastasis from lung cancer has been reported, but not from SCLC that is transformed from lung adenocarcinoma during maintenance treatment with epidermal growth factor receptor tyrosine kinase inhibitor (EGFR-TKI). Transformation to small cell lung cancer(SCLC), although uncommonly seen, has been associated with resistance to EGFR-TKI therapy in lung adenocarcinomas.

**Case presentation:**

We describe a case of a 49-year-old man with lung adenocarcinoma harboring L858R point mutation at the exon 21 of the epidermal growth factor receptor (EGFR). During the maintenance treatment with EGFR-TKI, the patient presented with a right breast mass, which was accompanied by elevated serum neuron specific enolase (NSE) level. The histological examination of biopsies from the breast mass and enlarging lung mass revealed SCLC that was less sensitive to standard SCLC treatment. The breast tumor was positive for thyroid transcription factor-1 (TTF-1), consistent with a lung primary cancer.

**Conclusion:**

This is the first case report of small cell transformation and metastatic to the breast in a patient with lung adenocarcinoma following EGFR-TKI treatment. Repeat biopsy is important for evaluation of evolving genetic and histologic changes and selection of appropriate treatment. and serum NSE measurement may be useful for detection of small cell transformation in cases with resistance to EGFR-TKI therapy.

## Background

Activating mutations in the epidermal growth factor receptor (EGFR) gene have been shown to be associated with a dramatic clinical response to EGFR tyrosine kinase inhibitors (EGFR-TKIs) in patients with lung adenocarcinomas [1, 2]. The strategy to preselect patients for this molecular based targeted therapy can increase the therapeutic response for patients with NSCLC [3, 4]. However, despite an initial response to the treatment with EGFR-TKIs, the majority of these patients eventually develop resistant to the drug treatment, a process termed acquired resistance [1]. Possible mechanisms of acquired resistance to EGFR-TKIs include secondary mutation in EGFR (T790M), MET amplification, over expression of hepatocyte growth factor (HGF), and loss of PTEN expression [1, 5–8]. In addition, tumor morphologic evolutions such as epithelial to mesenchymal transition and transformation to small cell lung cancer (SCLC), although uncommonly seen, have been associated with resistance to EGFR-TKI therapy in lung adenocarcinomas [9]. Herein, we report a case of SCLC transformation and metastasis to the breast in a patient with lung adenocarcinoma harboring EGFR mutation during the EGFR-TKI maintenance therapy. To our knowledge, this is the first report of breast metastasis from SCLC that is transformed from adenocarcinoma after EGFR-TKI treatment.

## Case presentation

**Fig. 1:**
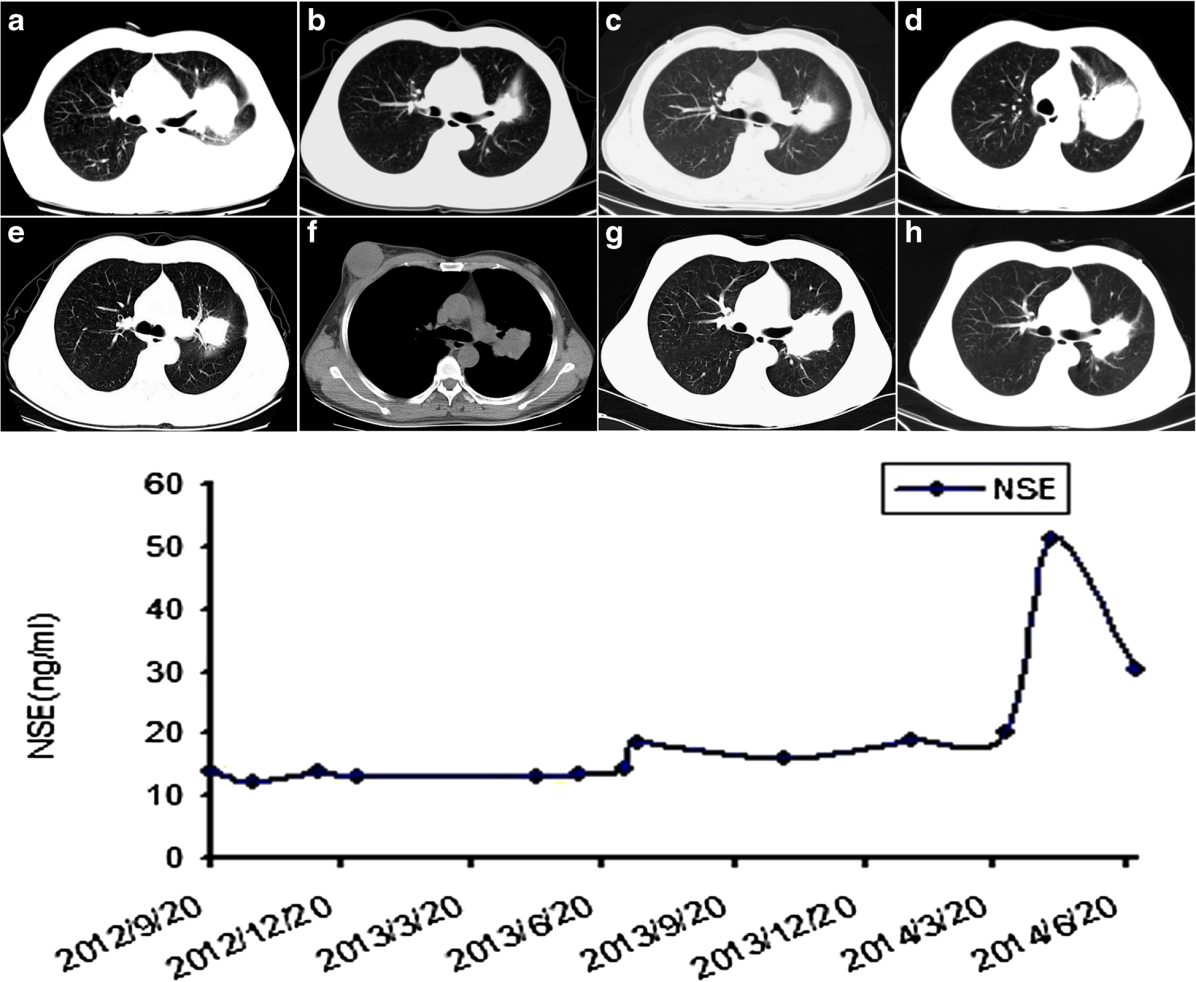
Computed Tomography (CT) Scans of the Case. (**a**) Chest CT Scan Showed a Mass in the Left Lingular Segment and Pleural Effusion Before Chemotherapy (September 2012). (**b**) Chest CT Scan Performed After 4 Cycles of Chemotherapy With Cisplatin and Pemetrexed (December 2012). (**c**) Chest CT Scan Performed after 4 Months of Treatment With Gefitinib (May 2013). (**d**) Chest CT Scan Performed After 6 Months of Treatment With Gefitinib (July 2013). (**e**) Evaluation Performed After 4 Cycles Chemotherapy With Cisplatin and Docetaxel in Addition to Gefitinib (November 2013). (**f**) Chest CT Scan Showed Right Breast Mass (March 2014). (**g**) Chest CT Showed Enlarging Lung Mass (March 2014). (**h**) Chest CT Evaluation Performed After 2 Cycles Chemotherapy With Cisplatin and Etoposide (June 2014)

**Fig. 2:**
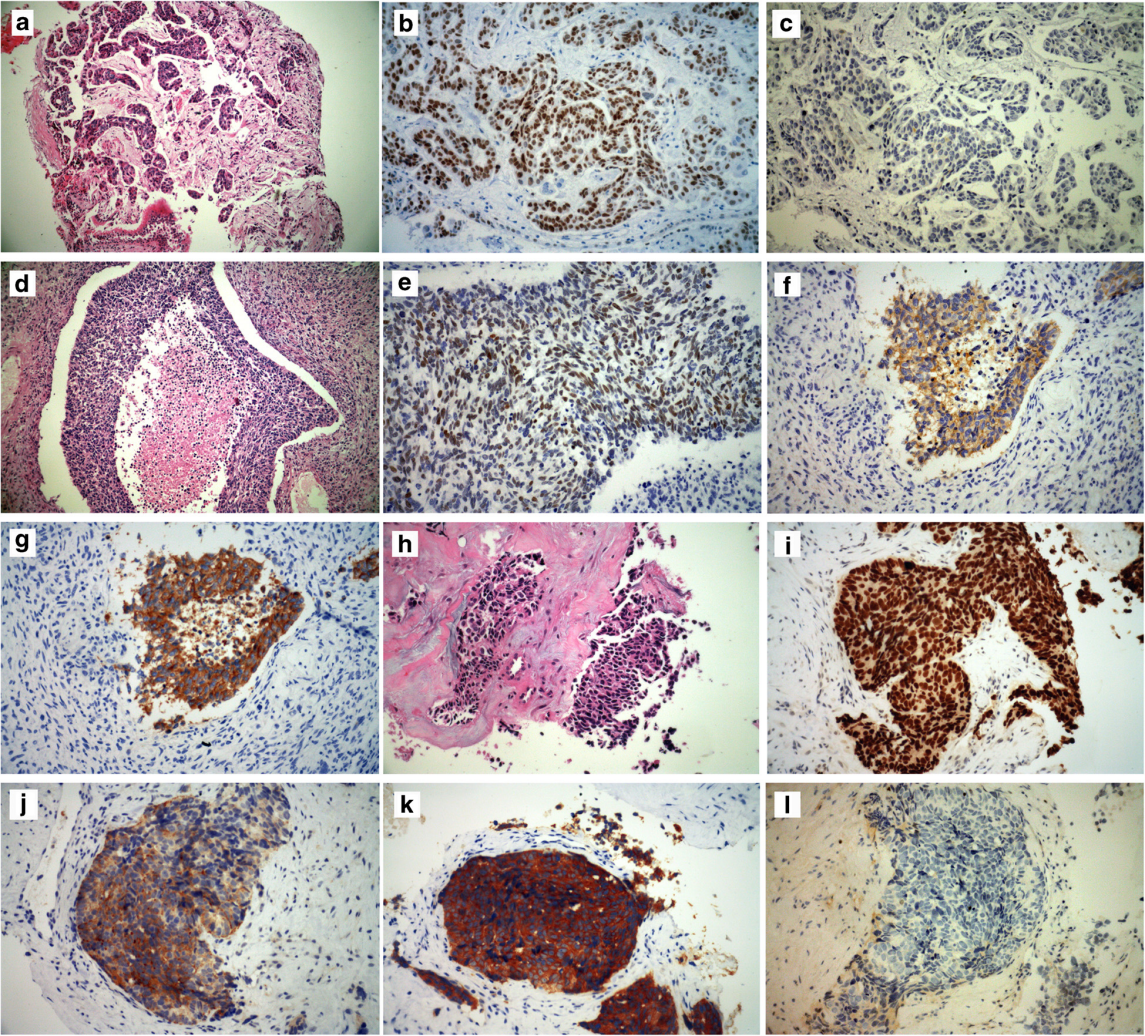
Hematoxylin–Eosin (HE) Staining of a Primary Lung Biopsy Specimen Revealed Adenocarcinoma(September 2012) (**a**). That Was Positive For TTF-1 (**b**) and Negative For Synaptophysin (**c**). Hematoxylin–Eosin Staining of a Secondary Biopsy Specimen in the Left Lingular Segment (July 2013) (**d**). Immunohistochemistry (IHC) Staining Showed High Expression of TTF-1 ( July 2013) (**e**). That Was Positive for Synaptophysin (**g**) and CD56 (**f**) (March 2014). Breast Mass Biopsy Specimens (X400) HE Staining Showed Small Cell Cancer Feature (March 2014) (**h**). Immunohistochemistry (IHC) Staining Showed High Expression of TTF-1 (**i**), Chomogranin A (**j**) and Synaptophysin (**k**), and a Negative Expression of estrogen receptor (ER) (**l**)

A 49-year-old man with 20 years’ history of smoking presented with cough and shortness of breath in September 2012. Chest computed tomography (CT) scan revealed a mass in the lingular segment of the left lung with mediastinal lymphadenopathy and moderate left pleural effusion (Fig. 1a). Serum tumor markers were elevated including CEA: 101 ng/mL (normal range, 0-5 ng/mL), CA19-9: 4018.2 u/L (normal range, 0- 35u/L), CYFRA21-1: 3.3 ng/mL (normal range, 0-2.0 ng/mL), but NSE: 13.8 ng/ml was in normal range (normal range, 0-14 ng/ml). Bronchoscopy examination showed bronchial narrowing/obstruction in the lingular segment, the biopsy of which confirmed adenocarcinoma of the lung (Fig. 2a–c). The cytological examination of pleural effusion was positive for malignant cells. The patient was staged as a stage IV tumor (cT2a, N2, M1a). The patient received four cycles of chemotherapy with cisplatin and pemetrexed and his symptoms including cough and dyspnea gradually improved. The tumor response was evaluated and considered as partial response in December 2012 (PR) (Fig. 1b). EGFR mutational analysis performed on the lung biopsy specimen revealed a L858R mutation in the exon 21 of EGFR. According to the NCCN guideline, gefitinib was given for maintenance therapy started in January 2013. The patient remained asymptomatic and the lung mass was stable until May 2013 (Fig. 1c) when the lung tumor started to grow slowly. In July 2013, repeat CT scan demonstrated that tumor increased its size (Fig. 1d). Serum tumor markers were then measured with the following results: CEA, 11 ng/mL; CA19-9, 10.8 u/L; and NSE, 14.3 ng/mL. Repeat biopsy of the re-growing lung mass was performed, which showed poorly differentiated carcinoma (Fig. 2d, e). Repeat EGFR mutational analysis revealed the same exon 21 mutation without additional mutations including T790M mutation. Two weeks later, serum tumor markers NSE were elevated (Fig. 1). In addition to the gefitinib the patient received four cycles of chemotherapy with cisplatin and docetaxel, which resulted in a partial response (PR) (Fig. 1e). He was followed up and received gefitinib treatment alone (250 mg daily). In March 2014, the patient complained of his right breast enlargement. Physical examination and chest CT scan revealed a 5-cm firm round mobile mass in his right breast at the 3 o’clock position (Fig. 1f). The breast mass was biopsied and showed poorly differentiated carcinoma with positive immunostaining for chomogranin A, synaptophysin, CD56, TTF-1 and negative for estrogen receptor(ER), GCDFP-15, HER2 (Fig. 2h, l). The second biopsy specimen from lingular segment was reassessed, confirming that the lung tumor was also positive for synaptophysin and CD56 (Fig. 2f, g). Thus, a diagnosis of metastatic small cell lung cancer (SCLC) was rendered for the breast tumor based on the morphologic and immunophenotypic features. The breast tumor also harbored the same EGFR exon 21 mutation. Repeat serum tumor marker test revealed that the level of NSE was increased to 51.2 ng/mL. Repeat CT scan showed lung mass enlargement and new multiple liver masses, considered as liver metastasis (Fig. 1g). Overall, the patient was considered to have acquired resistance to EGFR-TKI and transformation to SCLC. Gefitinib was discontinued and chemotherapy with regimen of cisplatin and etoposide was given. The patient showed initial clinical responses including shrinkage of lung and right breast tumors and the level of NSE decreased to 30.1 ng/mL (Fig. 1h). Unfortunately, the patient declined to have further treatment after he received six cycles of chemotherapy. Shortly after that, the patient developed bone and brain metastasis and died in Nov 2014.

## Discussion

The incidence of metastatic lung cancer to the breast is very low (<0.5 % of all metastatic disease) [10]. Mirrielees et al. [11] recently performed a systematic review of the literature and identified 41 independent case reports of primary lung carcinoma with metastasis to the breast. Of these case reports, 10 cases were SCLC and the remaining 31 cases were non-small cell lung cancer (NSCLC). The morphologic distinction between metastasis from primary lung cancer and primary breast cancer may be difficult. Immunohistochemical studies could be crucial for rendering a correct diagnosis. TTF-1 is a well-established biomarker for the tumor derived from the lung, and in our presented case, TTF-1 was expressed in the breast mass, consistent with a metastasis of lung primary. In Mirrielees report, TTF-1 was found to be expressed in 58 % of all NSCLC breast metastases and 83 % of those lung adenocarcinomas. All SCLC patients with breast metastasis were reported deceased within 8 months after the diagnosis. In contrast, 10 of 18 NSCLC patients with breast metastasis were alive for more than 2 years. Our case represented a breast metastasis of SCLC transformed from lung adenocarcinoma when developing acquired resistance to EGFR-TKI treatment. To our knowledge, this is the first report in the literature.

Transformation to SCLC following EGFR-TKI treatment is uncommon. To date, there are only a few case reports or small series. Zakowski et al first described a case of a 45-year-old female non-smoker with lung adenocarcinoma that was transformed to SCLC after acquired resistance to EGFR TKI therapy [9]. The patient expired after 7-month treatment with Etoposide. Morinaga et al. [12] and Watanabe et al. [13] later reported two similar cases: a 46-year-old female non-smoker with lung adenocarcinoma that transformed to SCLC 2 years after gefitinib treatment and a 52-year-old woman with lung adenocarcinoma that was transformed to SCLC 11 months after erlotinib treatment. Accumulating data have demonstrated that histological transformation to SCLC can occur in 4-14 % of EGFR-mutant NSCLC patients with acquired EGFR-TKI resistance [14, 15]. The mechanisms underlying transformation to SCLC are yet to be delineated. Two possible mechanisms have been postulated. First, a phenotype switch from NSCLC to SCLC may occur when the tumor acquires additional molecular alterations. And the other possibility is preexistence of mixed SCLC and adenocarcinoma, with SCLC becoming dominant after EGFR-TKI therapy [16]. In addition to the present case, 9 other cases of transformation to SCLC have been reported in the literature (Table 1) [9–13]. All the patients were female and smokers or with unknown history of smoking, and had a median age of 56.2 years (range, 36–67). However, our patient was male and he was a smokers. All the patients had documented activating EGFR mutations on the specimens of primary or/and repeat biopsies. In 7 patients who had EGFR mutation analysis performed on both primary and repeat biopsies, the EGFR mutations were identical. One patient (case 7) had an additional PIK3CA mutation in the repeat biopsy besides the EGFR L858R mutation. Nine patients received standard chemotherapy for SCLC, which resulted in responses in 7 cases. In clinical practice, tumor markers are often used to help diagnosis and monitor the disease. Watanabe et al reported changes in tumor markers when the tumor was transformed to SCLC; serum ProGRP and NSE levels were within normal limits prior to EGFR-TKI treatment, increased at the time of the repeat biopsy, and decreased following additional treatment [13] .Our case also showed similar changes in the level of NSE (Fig. 1). ProGRP and NSE may therefore be useful for the detection of SCLC transformation in patients developed resistance to EGFR-TKI treatment. Repeat biopsy should be considered in patients who show elevated those serum markers. However, additional studies with more patients and prospective series are required to confirm the usefulness of these tumor markers in monitoring progression of NSCLC during the treatment.

**Table 1:** Literature review of clinical and pathologic characteristics of lung adenocarcinomas with small cell carcinoma transformation

Case	Age	Sex	Smoking	Biopsy1	Genotype	Treatment	Biopsy2	Genotype	Reference
1	45	F	Never	Adeno	ND	Erlotinin	SCLC	19del	[9]
2	46	F	Never	Adeno	19del	Gefitinib	SCLC	19del	[10]
3	52	F	Never	Adeno	19del	Erlotinin	SCLC	19del	[11]
4	36	F	Never	Adeno	L858R	Geftinib	SCLC/Adeno	ND	[12]
5	67	F	ND	Adeno	L858R	EGFR-TKI	SCLC	L858R	[13]
6	54	F	ND	Adeno	19del	EGFR-TKI	SCLC	19del	[13]
7	56	F	ND	Adeno	L858R	EGFR-TKI	SCLC	L858R, PIK3CA	[13]
8	40	F	ND	Adeno	19del	EGFR-TKI	SCLC	19del	[13]
9	61	F	ND	Adeno	L858R	EGFR-TKI	SCLC	L858R	[13]
10	49	M	Yes	Adeno	L858R	Geftinib	SCLC	L858R	Our

ND: not-determined; Adeno: adenocarcinoma; 19del: EGFR exon 19 deletion; L858R: point mutation of EGFR exon 21; SCLC: small cell lung cancer; EGFR-TKI: epidermal growth factor receptor tyrosine kinase inhibitor

## Conclusion

We here reported a rare case of the patient who had small cell transformation and breast metastasis following EGFR-TKI treatment. Immunophenotypic analysis including TTF-1 is crucial to establish the diagnosis of breast metastasis from primary lung cancer. Measurement of serum NSE level may be helpful for suggesting small cell transformation. Repeat biopsy is important to enable histological and molecular analysis and guide appropriate treatment.

## Abbreviations

EGFR-TKI, Epidermal growth factor receptor tyrosine kinase inhibitor; ER, Estrogen receptor; HGF, Hepatocyte growth factor; NSCLC, Non-small cell lung cancer; NSE, Neuronspecific enolase; ProGRP, Pro-Gastrin-releasing peptide; SCLC Small cell lung cancer
